# Supplementary material for: Resolving the full spectrum of human genome variation using Linked-Reads
Source: Genome Res. 2019 Apr;29(4):635–45. doi: 10.1101/gr.234443.118 (PMC6442396; doi:10.1101/gr.234443.118)
Supplement: Supplemental Material [file supp_gr.234443.118_Supplemental_longranger-2.2.zip › longranger-2.2.tar.bz2!/lr2.2-public/lib/stan/cmdstan/stan/doc/api/html/chainable_8hpp_source.html]

Stan: src/stan/agrad/rev/chainable.hpp Source File


|  |  |
| --- | --- |
|  | Stan  2.5.0  probability, sampling & optimization |


- Main Page
- Related Pages
- Namespaces
- Classes
- Files

- File List
- File Members

All Classes Namespaces Files Functions Variables Typedefs Enumerations Enumerator Friends Macros Pages

- src
- stan
- agrad
- rev

chainable.hpp

Go to the documentation of this file.

1 #ifndef STAN\_\_AGRAD\_\_REV\_\_CHAINABLE\_HPP

2 #define STAN\_\_AGRAD\_\_REV\_\_CHAINABLE\_HPP

3

4 #include <vector>

5 #include <stan/agrad/rev/var\_stack.hpp>

6

7 namespace stan {

8  namespace agrad {

9

14  class chainable {

15

16  public:

17

22  chainable() { }

23

28  virtual ~chainable() {

29  // handled automatically

30  }

31

37  virtual void chain() {

38  }

39

44  virtual void init\_dependent() {

45  }

46

51  virtual void set\_zero\_adjoint() {

52  }

53

63  static inline void\* operator new(size\_t nbytes) {

64  return memalloc\_.alloc(nbytes);

65  }

66

77  static inline void operator delete(void\* /\* ignore arg \*/) {

78  /\* no op \*/

79  }

80  };

81

82

83

87  static void set\_zero\_all\_adjoints() {

88  for (size\_t i = 0; i < var\_stack\_.size(); ++i)

89  var\_stack\_[i]->set\_zero\_adjoint();

90  for (size\_t i = 0; i < var\_nochain\_stack\_.size(); ++i)

91  var\_nochain\_stack\_[i]->set\_zero\_adjoint();

92  }

93

110  static void grad(chainable\* vi) {

111

112  // simple reference implementation (intended as doc):

113  // vi->init\_dependent();

114  // size\_t end = var\_stack\_.size();

115  // size\_t begin = empty\_nested() ? 0 : end - nested\_size();

116  // for (size\_t i = end; --i > begin; )

117  // var\_stack\_[i]->chain();

118

119  typedef std::vector<chainable\*>::reverse\_iterator it\_t;

120  vi->init\_dependent();

121  it\_t begin = var\_stack\_.rbegin();

122  it\_t end = empty\_nested() ? var\_stack\_.rend() : begin + nested\_size();

123  for (it\_t it = begin; it < end; ++it) {

124  (\*it)->chain();

125  }

126  }

127

128  }

129 }

130 #endif

stan::agrad::chainable::init\_dependent

virtual void init\_dependent()

Initialize this chainable's adjoint value to make it the dependent variable in a gradient calculation...

**Definition:** chainable.hpp:44

var\_stack.hpp

stan::agrad::set\_zero\_all\_adjoints

static void set\_zero\_all\_adjoints()

Reset all adjoint values in the stack to zero.

**Definition:** chainable.hpp:87

stan::agrad::chainable

Abstract base class for variable implementations that handles memory management and applying the chai...

**Definition:** chainable.hpp:14

stan::agrad::memalloc\_

memory::stack\_alloc memalloc\_

**Definition:** var\_stack.cpp:16

stan::agrad::grad

static void grad(chainable \*vi)

Compute the gradient for all variables starting from the specified root variable implementation.

**Definition:** chainable.hpp:110

stan::agrad::nested\_size

static size\_t nested\_size()

**Definition:** var\_stack.hpp:103

stan::agrad::chainable::chain

virtual void chain()

Apply the chain rule to this variable based on the variables on which it depends. ...

**Definition:** chainable.hpp:37

stan::agrad::chainable::~chainable

virtual ~chainable()

Chainables are not destructible and should go on the function call stack or be allocated with operato...

**Definition:** chainable.hpp:28

stan::agrad::chainable::chainable

chainable()

Construct a chainable object.

**Definition:** chainable.hpp:22

stan::agrad::var\_stack\_

std::vector< chainable \* > var\_stack\_

**Definition:** var\_stack.cpp:13

stan::memory::stack\_alloc::alloc

void \* alloc(size\_t len)

Return a newly allocated block of memory of the appropriate size managed by the stack allocator...

**Definition:** stack\_alloc.hpp:168

stan::agrad::chainable::set\_zero\_adjoint

virtual void set\_zero\_adjoint()

Set the value of the adjoint for this chainable to its initial value.

**Definition:** chainable.hpp:51

stan::agrad::empty\_nested

static bool empty\_nested()

Return true if there is no nested autodiff being executed.

**Definition:** var\_stack.hpp:42

stan::agrad::var\_nochain\_stack\_

std::vector< chainable \* > var\_nochain\_stack\_

**Definition:** var\_stack.cpp:14


---

[ Stan Home Page ]

*© 2011–2014,
Stan Development Team.*
